# Clinical and radiographic results of lunate resection and vascularized os pisiform transfer for Kienböck's disease

**DOI:** 10.1016/j.jpra.2023.12.010

**Published:** 2023-12-15

**Authors:** Masaomi Saeki, Hidemasa Yoneda, Michiro Yamamoto

**Affiliations:** Department of Human Enhancement & Hand Surgery, Nagoya University Graduate School of Medicine, Nagoya, Japan

**Keywords:** Postoperative, Kienböck's disease, Pisiform, Vascularized bone graft

## Abstract

Although various treatments for advanced stages of Kienböck's disease have been reported, clinical evidence demonstrating the efficacy of lunate resection and vascularized os pisiform transfer for Kienböck's disease is limited. Herein, we investigated the clinical and radiographic results of this procedure. We retrospectively investigated eight patients who were followed up for ≥1 year. The mean age at the time of surgery was 52 years. The mean follow-up period was 3.4 years. The preoperative and postoperative mean wrist flexion–extension ranges were 84° and 111°, respectively, and grip strengths were 18.5 and 26.3 kg, respectively. Pain decreased in five patients postoperatively. The mean preoperative and postoperative carpal height ratios were 0.47 and 0.46, respectively, and radio scaphoid angles were 63° and 65°, respectively. Osteoarthritic changes were observed in or around the transferred pisiform in all five patients who were surveyed using radiographs. Most patients demonstrated satisfactory clinical results, including pain relief and improved wrist motion and grip strength, regardless of osteoarthritic wrist changes on postoperative radiographs. In summary, this procedure was effective for treating Kienböck's disease, especially in the advanced stages.

Level of evidence: Ⅳ

## Introduction

Various treatments for advanced stages of Kienböck's disease have been reported, including partial carpal fusions in the form of scaphotrapezium-trapezoid or scaphocapitate arthrodeses, proximal low carpectomy, and lunate replacement.^1^, ^2^, ^3^, ^4^ Lunate resection and vascularized os pisiform transfer for advanced stages of Kienböck's disease was first reported by Saffar in 1982.^5^ Some case series have reported that lunate resection and vascularized os pisiform transfer yield favorable clinical results, as reflected by pain relief and improved range of motion (ROM). In contrast, radiographic findings such as significantly reduced pisiform bone and carpal collapse progression have been reported.^6^^,^^7^

There is a limited number of published studies concerning lunate resection and vascularized os pisiform transfer for advanced stages of Kienböck's disease, primarily because of the disease's rarity and the limited number of patients who have undergone this surgery. Based on the currently available reports, reaching a consensus on the clinical and radiographic results of this procedure is difficult. This study aimed to investigate the clinical and radiographic results of lunate resection and vascularized os pisiform transfer in patients with Kienböck's disease.

## Methods

### Patient characteristics

The study was approved by the authors’ home institution's ethics committee (approval number: 2018–0007) and followed the principles of the Declaration of Helsinki. Ten patients from two institutes who underwent lunate resection and vascularized os pisiform transfer for Kienböck's disease between April 2011 and April 2022 were retrospectively assessed. Eight of the ten patients (five men and three women) who could be followed up for at least 1 year were included in this study. Patient ages ranged from 18 to 78 years (mean [standard deviation (SD)], 52 [20] years) at the time of surgery. Three patients were housewives, one was unemployed, one worked as a sake (wine) dealer, one as a train mechanic (regularly using hand tools), one as a transport worker, and one was a student (practicing judo). The right wrist was affected in six patients and the left wrist in two patients. Using conventional radiographic evaluation, seven patients were classified as having Lichtman stage IIIb and one as Lichtman stage II.^8^^,^^9^ The postoperative observation period ranged from 1 year to 9 years and 2 months [mean (SD), 3.4 (2.6) years; Table 1].

**Table 1:** Characteristics of patients who underwent vascularized os pisiform transfer in Kienböck's disease.

Case no.	Age (years)	Sex	Occupation	Affected side	Lichtmann's stage	Period since operation(y: years, m: months)
1	70	F	Housework	Left	II	1 y, 0 m
2	78	M	Unemployed	Right	IIIb	1 y, 0 m
3	54	M	Sake (wine) dealer	Right	IIIb	3 y, 6 m
4	37	M	Train mechanic (uses a hammer)	Right	IIIb	9 y, 2 m
5	67	F	Housework	Right	IIIb	8 y, 0 m
6	44	M	Transport worker	Left	IIIb	5 y, 8 m
7	18	M	Student(practicing judo)	Right	IIIb	3 y, 0 m
8	47	F	Housework	Right	IIIb	1 y, 11 m

F: female, M: male.

### Surgical technique

**Figure 1:**
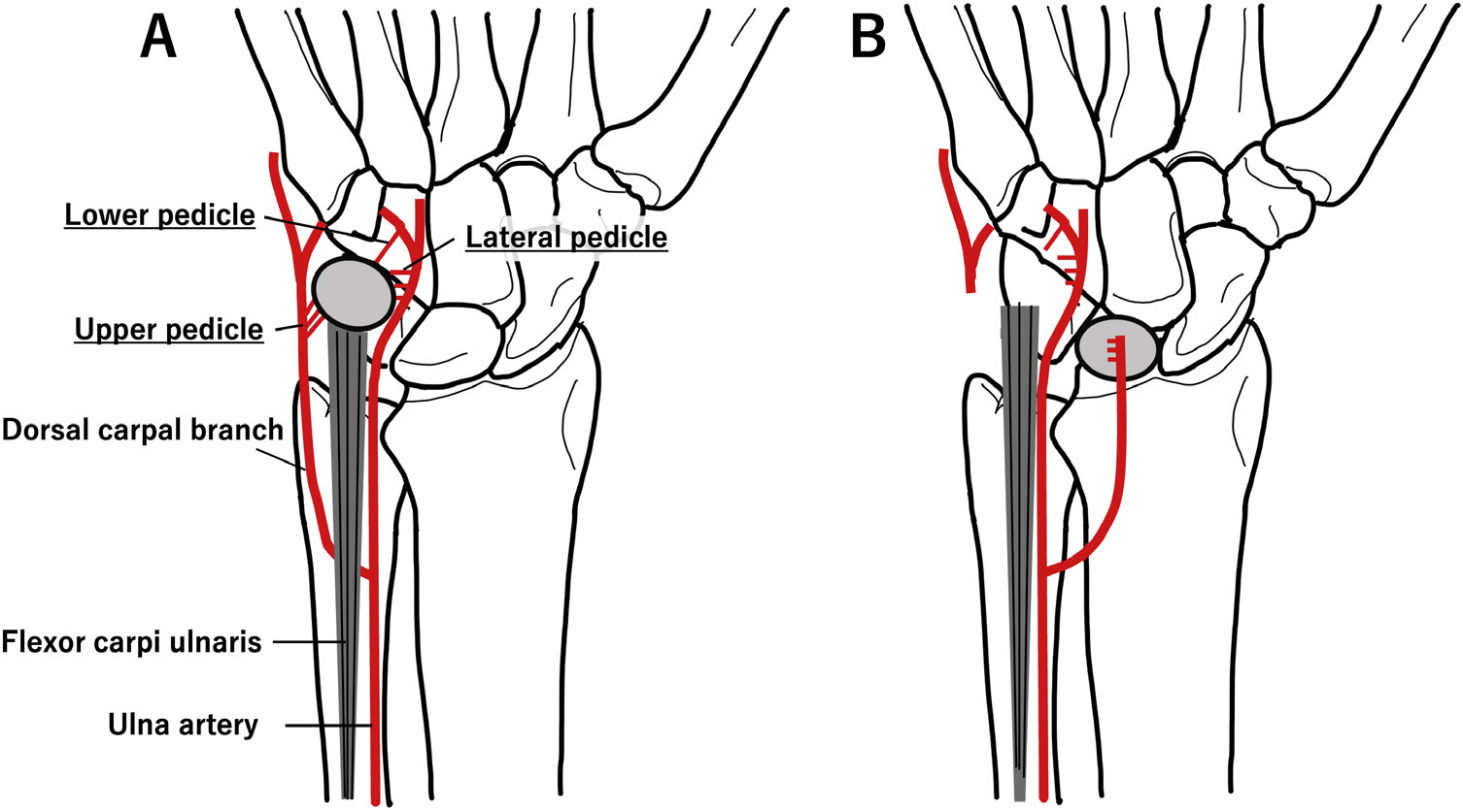
A) Surgical anatomy. The blood supply to the pisiform bone is provided by three pedicles: an upper pedicle arising from the dorsal carpal branch of the ulnar artery, a lateral pedicle arising directly from the ulnar artery, and a lower pedicle arising from the deep palmar branch of the ulnar artery. B) Surgical procedure. The pisiform was dissected from the muscular attachment and the flexor carpi ulnaris tendon. The site where the vascular pedicle entered the pisiform bone from the side of the pisotriquetral joint was retained, and the pisiform bone was elevated. The pisiform was inserted into the void resulting from the excision of the lunate bone.

In this study, all patients underwent surgery, which was performed by specialists certified by the Japanese Society for Surgery of the Hand. The surgeon determined the surgical technique. The surgery was performed under regional anesthesia by brachial plexus block in all eight patients, and tourniquets were used. The procedures for elevating the vascularized pisiform followed a technique previously described by our group, which followed the original description by Saffar.^5^^,^^10^ A longitudinal palmar incision of the forearm was made along the flexor carpi ulnaris tendon and was extended distally in a zigzag fashion at the site of the wrist crease. The pisiform was dissected from the muscular attachment and the flexor carpi ulnaris tendon. The vascular pedicle was separated from the dorsal branch of the ulnar nerve along which it ran. The site where the vascular pedicle entered the pisiform bone from the side of the pisotriquetral joint was retained, and the pisiform bone was elevated. The tourniquet was released, and blood flow to the pisiform bone was confirmed. The carpal tunnel was opened, and the flexor tendons and median nerve were retracted radially. The anterior capsule was opened using a U-shaped incision. The pisiform was inserted into the space created by the excision of the lunate. Temporary fixation of the grafted bone with K-wires was performed in three patients. After the operation, external fixation was applied to all patients for approximately 4 weeks. In most cases, physiotherapy began after the removal of the external fixation (Figure 1).

### Data assessment

Clinical and imaging data were obtained from hospital records. At the follow-up examination, the patient's pain was assessed on four levels (no pain, mild, moderate, and severe), as indicated on medical records. Clinical assessment at the follow-up examination included the ROM of the wrists measured using a goniometer and grip strength measured using a dynamometer. The postoperative results were evaluated using Hand20, an illustrated questionnaire developed by our group, and its abbreviated version, Hand10.^11^, ^12^, ^13^

Radiographs of the wrist in the standard posteroanterior and lateral views were obtained at the follow-up examination for all patients and compared with the preoperative and postoperative radiographs. Radiographs were evaluated to determine the stage of Kienböck's disease according to Lichtman's criterion, changes in the carpal height ratio (CHR) as determined by Youm et al.,^14^ and the radio scaphoid angle (RSA). The postoperative Hand20 score was analyzed in four patients but not in the remaining two patients because of incomplete documentation. Radiographs of the unaffected side were assessed in five patients. Other survey items were obtained from all six patients. We also assessed the satisfactory results using Lichtmann's criterion.^15^

### Statistical analyses

Between-group differences for continuous variables were evaluated using Wilcoxon signed-rank test. P-values of <0.05 were considered statistically significant. We calculated the preoperative CHR and RSA by comparing the affected and unaffected sides and the CHR and RSA on the affected side was calculated by comparing the preoperative and postoperative values.

## Results

At the follow-up evaluations, pain decreased in seven patients. Three patients who had preoperatively severe or moderate pain were postoperatively free of pain. In the other four patients, preoperative moderate or severe pain became mild postoperatively. The other patient's pain was mild and did not change after surgery. In seven patients, ROM improved after surgery, postoperative wrist flexion and extension ranged from 75° to 140° [mean (SD), 111° (20°)], and preoperative values ranged from 59° to 130° [mean (SD), 84° (25°)]. Postoperatively, ROM decreased in one patient; flexion was 60° preoperatively and 50° postoperatively and extension was 70° preoperatively and 55° postoperatively at 5-year and 8-month follow-ups. The mean (SD) preoperative and postoperative grip strengths were 18.5 (9.3) and 26.3 (11.3) kg, respectively. Grip strength increased postoperatively in seven patients and decreased in one patient. Postoperative grip strength ranged from 42% to 121% [mean (SD), 86.5% (23.9%)] of the contralateral side. At follow-up evaluation, the Hand20 scores (possible range 0–100) ranged from 0 to 47 [mean (SD), 21.4 (22.2)]. In the current series, six of eight patients had a satisfactory rating according to Lichtmann's criterion. The reasons for the unsatisfactory rating, according to Lichtmann's criterion, were low grip strength in one patient and decreased ROM in another patient (Table 2).

**Table 2:** Clinical outcomes of patients who underwent vascularized os pisiform transfer in Kienböck's disease.

Case no.	Range of motion (Flexion/Extension)		Grip strength		Postoperative strength (% of the unaffected side)	Pain		Hand20 (postoperative)	Result
	Preoperative (°)	Postoperative (°)	Preoperative (kg)	Postoperative (kg)		Preoperative	Postoperative		
1	50/45	50/76	14	10	42	Severe	Mild	43.5	Unsatisfactory
2	60/35	70/45	9	23	121	Severe	No	—	Satisfactory
3	50/50	70/55	19	36	63	Moderate	No	—	Satisfactory
4	50/45	75/65	34	36	92	Moderate	No	5.5	Satisfactory
5	50/30	60/40	8	21	95	Severe	Mild	47	Satisfactory
6	60/70	55/50	30	31	94	Moderate	Mild	11	Unsatisfactory
7	35/25	65/40	19	32	98	Moderate	Mild	0	Satisfactory
8	30/30	55/20	15	20	87	Mild	Mild	—	Satisfactory

Postoperative results evaluated by Lichtmann's criteria of 1982.^16^

### Radiographic findings

The CHR and RSA of the affected side were examined in eight patients. The CHR and RSA of the unaffected side were examined in seven patients because radiographic examination of the unaffected side was not performed in one patient.

**Figure 2:**
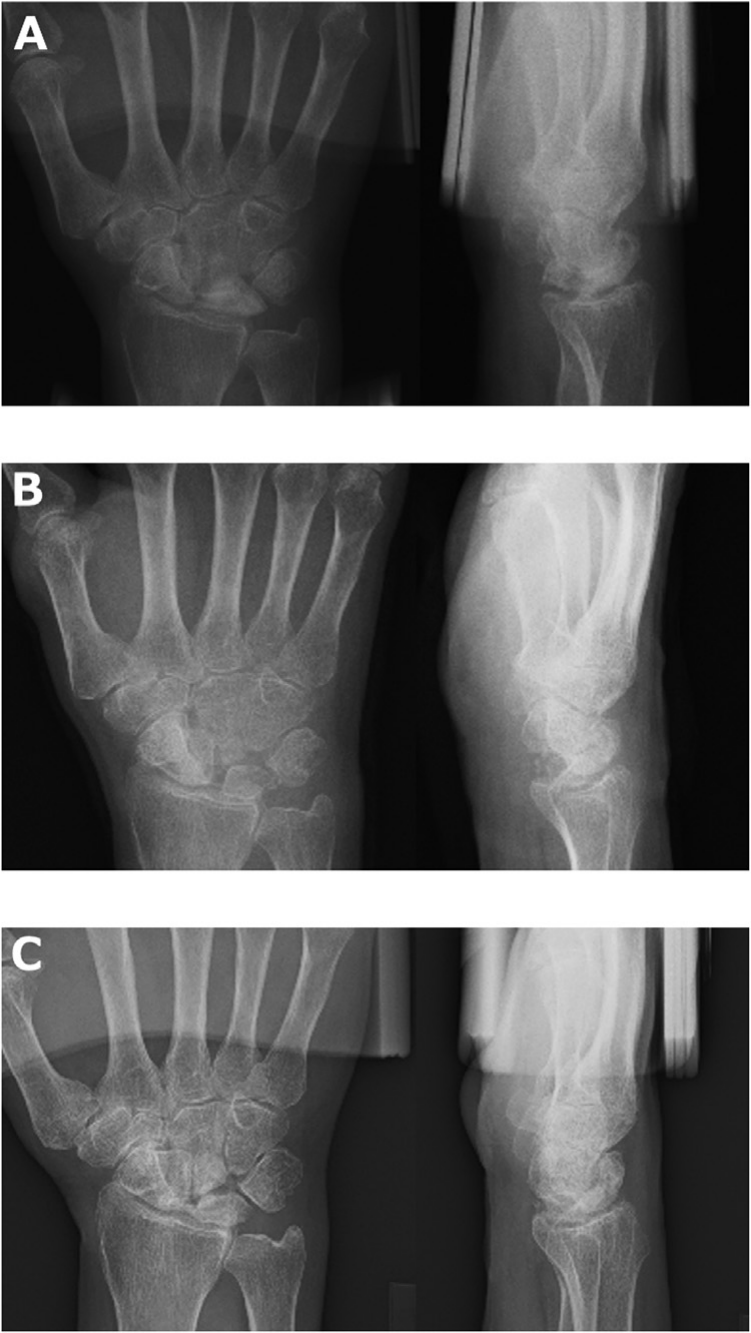
Radiograph of the right wrist of patient no. 5, a 67-year-old woman with stage IIIb Kienböck's disease. Six years and 1 month postoperatively, a radiographic change in and around the pisiform was found, especially between the capitate and the transferred pisiform. The patient experienced decreased pain, ranging from severe to mild at the last follow-up. A) Before surgery, B) immediately after surgery, C) 6 years and 1 month after surgery.

The mean (SD) preoperative CHR was 0.47 (0.04) on the affected side and 0.51 (0.04) on the unaffected side, which was significantly lower on the affected side (*P* = 0.047). The mean (SD) postoperative CHR on the affected side was 0.46 (0.03), which decreased from the preoperative value without statistical significance (*P* = 0.71). The mean (SD) RSA before surgery was 63° (10°) on the affected side and 55° (13°) on the unaffected side, and no significant difference was found (*P* = 0.11). The mean (SD) postoperative RSA on the affected side was 65° (10°), an increase from the preoperative value without statistical significance (*P* = 0.71). Osteoarthritic changes on radiography in the transferred pisiform and the bones around the transferred pisiform were found in five patients (Figure 2; Table 3).

**Table 3:** Radiographic findings of patients undergoing vascularized os pisiform transfer in Kienböck's disease.

CaseNo.	CHR			RSA			Radiographic changes in or around the transferred pisiform
	Affected side		Unaffected side	Affected side		Unaffected side	
	Preoperative	Postoperative		Preoperative (°)	Postoperative (°)		
1	0.51	0.50	0.54	50	55	62	−
2	0.44	0.44	0.56	65	58	44	−
3	0.47	0.47	—	62	59	—	−
4	0.39	0.40	0.44	82	85	76	○
5	0.49	0.46	0.52	60	72	61	○
6	0.46	0.46	0.47	67	64	56	○
7	0.47	0.49	0.53	63	70	48	○
8	0.51	0.49	0.53	52	58	37	○
Average	0.47±0.04	0.46±0.03	0.51±0.04	63±10	65±10	55±13	

CHR: carpal height ratio, RSA: radio scaphoid angle.

### Complications

There were no perioperative or postoperative complications. No further surgery was performed on any patients.

## Discussion

This study investigated postoperative outcomes after lunate resection and vascularized os pisiform transfer to treat Kienböck's disease in patients who were followed up for at least 1 year after surgery. The results showed that six out of eight cases were rated as satisfactory according to Lichtmann's criterion, and the CHR and RSA values at the last postoperative measurement showed no significant changes compared to their preoperative values. Osteoarthritic changes on radiographs in or around the transferred pisiform were observed in all patients evaluated using radiography.

Lunate resection and vascularized os pisiform transfer for advanced stages of Kienböck's disease did not produce good radiographic results. Radiographic findings such as significantly reduced pisiform bone, progression of carpal collapse, and osteoarthritis around the pisiform in the long term have been reported.^16^ Some clinical studies have reported the results of lunate resection and vascularized os pisiform transfer as a treatment for advanced stages of Kienböck's disease.^6^^,^^7^^,^^16^ A clinical study with a mean (SD) postoperative follow-up period of 9.9 (3.5) years showed an average decrease in Nattrass index scores and an increase of RSA values in approximately half of the patients with documented carpal deterioration; this was also true for five of the eight patients in our study.^17^ Another study with 11 patients reported decreased CHR and increased RSA values at the middle follow-up at 3 to 5 years postoperatively. However, there were no significant differences in the CHR and RSA values between the middle and final follow-up at an average of 19 years postoperatively.^6^ In contrast with these studies, the present study showed that CHR and RSA at the last postoperative measurement showed no significant changes compared with the preoperative measurements. This study's average (SD) postoperative follow-up period was 3.4 (2.6) years, and longer-term observations are required to evaluate the deterioration of CHR and RSA values.

A retrospective study investigated the results of vascularized os pisiform transfer and showed that osteoarthritis was found in 10 of 21 patients at follow-up evaluations.^17^ Another study showed that osteoarthritic changes in the wrist joint were found in 4 of 11 patients. In the present study, the osteoarthritic changes in or around the transferred pisiform were found in five of eight patients. As in previous studies, the postoperative osteoarthritic change in our study occurred in all cases where radiographic investigation was possible. A previous clinical study also reported a trend toward osteoarthritis in patients with greater preoperative RSA values.^17^ In this study, all patients had osteoarthritic changes, and the RSA values were relatively high (58–85°), although no comparisons were made between patients with and without osteoarthritis.

The cause of transferred pisiform deterioration is unclear. A previous retrospective clinical study reported that deterioration was most likely caused by compromised perfusion of the pisiform.^17^ Our previous investigation on the evaluation of transferred pisiform using magnetic resonance imaging revealed that the signal changes were confirmed in the opposite part of the bone around the pisiform in all included patients.^18^ Wrist osteoarthritis after lunate resection and vascularized os pisiform transfer for advanced stages of Kienböck's disease could be attributed to less congruency of the transferred pisiform and surrounding bones than the compromised perfusion of the pisiform.

In Saffar's original method, the cartilaginous surface of the pisiform bone is inserted in contact with the proximal or radial articular surface.^5^ The pisiform has cartilage only on the articular surface of the pisotriquetral joint and no cartilage on other surfaces. The volume of the pisiform is considerably smaller than that of the lunate.^19^ From this point of view, it seems reasonable that the grafted pisiform shows osteoarthritic changes between the surrounding bones. However, by including the degree of carpal collapse as a criterion for surgical indication or measuring the space created by the lunate excision and the size of the pisiform bone preoperatively, the higher congruency may contribute to reducing the incidence of transferred pisiform deterioration.

In the present study, six and two patients had satisfactory and unsatisfactory ratings according to Lichtman's criterion, respectively. In one patient for whom an unsatisfactory rating was caused by decreased ROM, postoperative progression of the deterioration in transferred pisiform and bones around the transferred pisiform was found to be remarkable.

In contrast, the transferred pisiform of one patient, as assessed 2 years and 5 months postoperatively, was fractured and split into two in the coronal plane. The follow-up clinical results showed that the patient's wrist pain had disappeared, preoperative condition had improved, and excursion and grasping power had also improved. Exactly when pisiform bone fractures occur after surgery remains unclear. The pisiform is a sesamoid bone; its kinematic characteristics are different from those of the other carpal bones and it is not in an environment of force transmission across the carpus. It is possible that the mechanism of the fracture was due to the magnitude and concentration of the load from the capitate as well as the strength of the pisiform.^20^

There are some limitations to this study. This was a retrospective clinical research study with a small sample size. The incidence of asymptomatic Kienböck's disease is <2%.^21^ The advanced stages of Kienböck's disease are treated using various surgeries, including wrist arthrodesis, proximal row carpectomy, and lunate replacement. Of these, the number of patients who undergo lunate resection and vascularized os pisiform transfer in Kienböck's disease is even smaller. The only studies reporting this treatment are limited by their small sample sizes. For evidence-based clinical practice in such rare cases, it is important to reach a consensus on this procedure's clinical and radiographic results using systematic research on clinical data from small-scale reports.

In summary, we performed a postoperative analysis of the transferred pisiform and surrounding bones in patients who underwent lunate resection and vascularized os pisiform transfer for the advanced stages of Kienböck's disease. Most patients demonstrated satisfactory clinical results, including pain relief and improved wrist ROM and grip strength. Nevertheless, wrist osteoarthritis after surgery was found in all patients who underwent postoperative radiographic examinations. This procedure is effective for treating Kienböck's disease, especially in the advanced stages.

## Declaration of competing interest

None.
